# Determination of Intra- and Extracellular Metabolic Adaptations of 3D Cell Cultures upon Challenges in Real-Time by NMR

**DOI:** 10.3390/ijms23126555

**Published:** 2022-06-12

**Authors:** Christian Urzì, Damian Hertig, Christoph Meyer, Sally Maddah, Jean-Marc Nuoffer, Peter Vermathen

**Affiliations:** 1 Departments of Biomedical Research and Neuroradiology, University of Bern, Hochschulstrasse 6, 3012 Bern, Switzerland; christian.urzi@students.unibe.ch (C.U.); damian.hertig@students.unibe.ch (D.H.); christoph.m.meyer@gmail.com (C.M.); s.maddah@enderdiagnostics.com (S.M.); 2 Department of Clinical Chemistry, University Hospital Bern, Freiburgstrasse, 3010 Bern, Switzerland; jean-marc.nuoffer@insel.ch; 3 Graduate School for Cellular and Biomedical Sciences, University of Bern, Mittelstrasse 43, 3012 Bern, Switzerland; 4 Department of Pediatric Endocrinology, Diabetology and Metabolism, University Children’s Hospital of Bern, Freiburgstrasse, 3010 Bern, Switzerland; 5 Translational Imaging Center (TIC), Swiss Institute for Translational and Entrepreneurial Medicine, 3010 Bern, Switzerland

**Keywords:** NMR, bioreactor, fibroblasts, metabolomic, diffusion, intracellular fingerprints, extracellular footprints

## Abstract

NMR flow devices provide longitudinal real-time quantitative metabolome characterisation of living cells. However, discrimination of intra- and extracellular contributions to the spectra represents a major challenge in metabolomic NMR studies. The present NMR study demonstrates the possibility to quantitatively measure both metabolic intracellular fingerprints and extracellular footprints on human control fibroblasts by using a commercially available flow tube system with a standard 5 mm NMR probe. We performed a comprehensive 3D cell culture system characterisation. Diffusion NMR was employed for intra- and extracellular metabolites separation. In addition, complementary extracellular footprints were determined. The implemented perfused NMR bioreactor system allowed the determination of 35 metabolites and intra- and extracellular separation of 19 metabolites based on diffusion rate differences. We show the reliability and sensitivity of NMR diffusion measurements to detect metabolite concentration changes in both intra- and extracellular compartments during perfusion with different selective culture media, and upon complex I inhibition with rotenone. We also demonstrate the sensitivity of extracellular footprints to determine metabolic variations at different flow rates. The current method is of potential use for the metabolomic characterisation of defect fibroblasts and for improving physiological comprehension.

## 1. Introduction

Mitochondria are involved in several metabolic signalling pathways, besides being the main cellular site for the production of ATP. Therefore, there is a strong interdependence between energy production and cellular metabolism. Respirometric analysis is well known for the characterisation of mitochondrial respiration, thus for the assessment of the aerobic energy production [1,2]. On the other hand, analytical methods such as high-resolution Nuclear Magnetic Resonance (NMR) and Mass Spectroscopy (MS) are widespread for the quantitative assessment of the metabolome [3,4,5,6,7]. We have previously shown the feasibility to measure in real-time mitochondrial respiration and metabolic changes of fibroblasts embedded in collagen-based 3D matrix by using a perfused NMR bioreactor system [8]. The reproducibility of metabolomic measurements on living cell cultures was assessed and the sensitivity of the instrument demonstrated to measure over time surrogate markers of major mitochondrial fuel pathways such as glycolysis, glutaminolysis, and mitochondrial oxidative phosphorylation (OXPHOS) by performing measurements with stopped and continued perfusion (“stop and go” measurements). We also showed the sensitivity of the setup to measure anaerobic glycolytic activity and basal oxygen consumption over time in continuous perfusion conditions upon inhibitor challenges. However, intracellular metabolism was not studied directly, since the obtained NMR spectra included both, cells and medium contributions, and the exact cellular volume and the ratio of cells to medium in the NMR sensitive volume of the bioreactor were unknown. A significant problem in metabolomic NMR studies of cells in culture is the discrimination of intra- and extracellular contributions to the spectra. This aspect was addressed in ex vivo studies of perfused cells in which the extracellular fraction predominates in terms of the occupied volume compared to the intracellular space [9], which makes intracellular metabolic changes more difficult to detect. Therefore, the capability to separately measure and combine the metabolic intracellular fingerprints and extracellular footprints of supernatants would allow for a better characterisation of ongoing bioenergetic and metabolic processes. Metabolic footprint analysis yields information about extracellular metabolites that are mainly produced or consumed as by-products of the cellular metabolic activity [10], and composition changes are due in response to different factors such as temperature, pH, oxygen availability, specific interventions, and strongly depend also on the media composition [1]. Because of the availability of different analytical tools and the ease of sample collection, extracellular supernatant analysis has been widely used in metabolomics [11], genomics [12,13], proteomics [14], and other fields. However, monitoring of extracellular metabolites only provides a complementary picture of what is going on intracellularly. For this reason, additional information on intracellular processes is required. In the current paper we used diffusion-weighted NMR spectroscopy in order to distinguish between intra- and extracellular contributions and to provide specific information about the intracellular fraction [9]. Extracellular molecules diffuse freely because they are not subjected to membranes or organelles shielding, and their diffusion decay is described by a relatively long diffusion constant. In contrast, the self-diffusion of compartmentalised molecules is attenuated by intracellular factors (i.e., cellular membranes, organelles, cytosol viscosity, and molecular crowding) and correspondingly their diffusion constant is short [9,15,16]. Therefore, the separation of fast from slow diffusing contributions allows the study of intra- and extracellular metabolite pools separately.

To our knowledge, no study has focused on intra- and extracellular real-time metabolic analysis of living cell cultures inside the NMR. The aims of this study are therefore:

1. To employ diffusion-weighted NMR spectroscopy to separate between intra- and extracellular metabolic contributions in living human 3D cell cultures in a perfused bioreactor system and to investigate in vitro intracellular metabolic fingerprints;
2. To collect from the bioreactor outflowing supernatant and to perform extracellular metabolic footprint characterisation providing complementary insights on the metabolic ongoing processes.

## 2. Results

**Figure 1:**
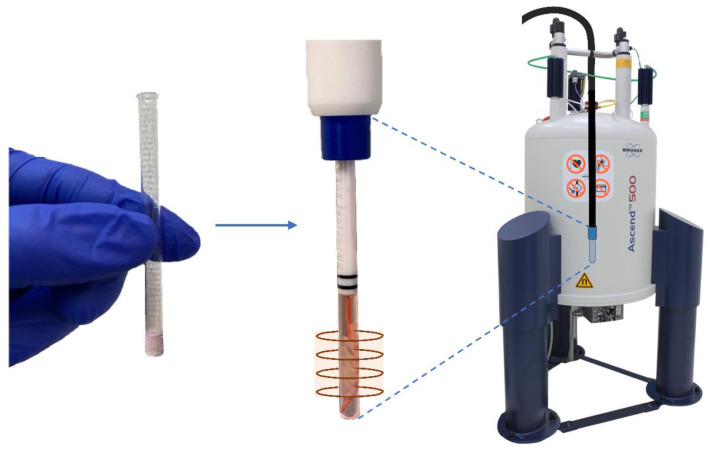
Insertion of 3D collagen-based sample embedded fibroblasts (FBCO04), clamped within the sensitive region of the NMR coil by a collagen-based plug placed at the bottom of the 5 mm NMR glass tube, inside the NMR spectrometer.

The previous initial study demonstrated in detail the successful implementation of a relatively simple and commercially available flow system employing standard 5 mm NMR tubes for metabolic investigations of living cells in real-time [8] (Figure 1). Based on these results the current paper describes several improvements and further developments: (I) The quality of ^1^H and ^31^P NMR spectra was improved (II) Water diffusion measurements and ^23^Na measurement were employed for cellular volume evaluation; most importantly, (III) Diffusion measurements were performed to separately determine intra- and extracellular metabolic fingerprints, and (IV) Cell footprints were determined simultaneously revealing the sensitivity to detect metabolic changes in the outflowing supernatant; (V) Finally, the applicability of the advancements was validated by showing the capability to determine intra- and extracellular metabolic adaptions upon inhibitors addition.

### 2.1. 3D Cell Culture Characterisation

#### 2.1.1. Evaluation of Scaffold Volume

We had previously observed a cell density dependent shrinkage of the scaffold volume especially within the first 24h [8], but it remained unclear whether the scaffold size reduction was due to either fibroblasts contractile action or degradation of the hydrogel. Here, we aimed at answering this via measurement of the ^23^Na signal, which stems mainly from the extracellular medium with high sodium content. Collagen degradation would lead to an increase in the ratio medium to scaffold in the sensitive region of the NMR coil, therefore to an increase in the ^23^Na signal; scaffold shrinkage (like a sponge) with no polymer chain degradation would not change this ratio, therefore non-significant sodium changes would be expected. Three different cell numbers, 5, 10, and 20 million fibroblasts, were embedded in 3D collagen-based scaffolds. For each of these cell numbers, four 3D samples were prepared in parallel and cultured in a standard cell culture incubator up to 24 h. During this period, each sample per cell number was observed with an optical microscope at specific time points (1, 6, 20, and 24 h) and subsequently measured with NMR for ^23^Na evaluation. The microscopy analysis confirmed the dependence of volume reduction on cell density, as it has been shown similarly before in [8], with a decrease down to 37% to 74% after cell encapsulation over a period of 24 h. The largest volume contraction occurred within the first 20 h, then only of 2–3% up to 24 h (Figure S1, see Supplementary Materials). ^23^Na NMR measurements were performed on the same samples, at the same time points. Sodium signal values normalised to the sum of all ^23^Na measurements are reported in Figure S1. In contrast to the strong volume shrinkage over time no significant sodium signal change was detected, neither over time nor for different cell densities. This reveals that reduction in volume was mainly due to fibroblast mediated contraction rather than degradation of 3D collagen-based matrix in standard cell culture incubator before NMR measurements. The large experimental error up to 14%, calculated for each cell density from the normalised ^23^Na value at time point 1h, was probably due to the scaffolds’ weakness at the first time points with loss of small fragments during sample placement into the standard 5 mm NMR tube. Therefore, contraction of collagen embedded fibroblasts led to volume reduction and stabilisation, and to an increase in scaffold strength. Based on these results, all subsequent NMR measurements in the bioreactor were performed after 24 h from 3D sample realisation under stable scaffold volume conditions, in order to obtain reproducible data in repeated experiments.

#### 2.1.2. Cellular Volume Evaluation with ^23^Na NMR

^23^Na NMR spectra of perfused 3D collagen-based matrix with 10 million fibroblasts were also used for cellular volume determination. The same measurement was performed on cell culture medium in a regular 5 mm NMR glass tube in order to obtain a reference value without cells and scaffold. Considering an extracellular sodium concentration of 144.4 mM of standard cell culture minimal essential medium (MEM) [17,18] and an intracellular human fibroblasts sodium concentration of 10 mM [19], the percentage cell volume was calculated according to [20]. The analysis revealed that 3D collagen-based embedded cells occupy 1.43% of the total sample volume within the sensitive NMR coil. By considering the sample volume within the sensitive NMR region, which was estimated to amount to 243 μL and approximating the volume of a single cell as a sphere, we found an average radius of 4.36 μm for embedded fibroblasts, in agreement with values found in [21,22]. Furthermore, the same measurement performed on a sample with 20 million fibroblasts led to a consistent value of 2.9%.

#### 2.1.3. Cell Viability

Supernatant samples were collected at the beginning and at the end of every measurement, and lactate dehydrogenase (LDH) activity was evaluated as described in [8] using Roche diagnostics cobas 8000 analyser. No increase in LDH activity was encountered in any of the performed bioreactor measurements, thus excluding cytolysis. Cell well-being was additionally monitored in most experiments by performing ^31^P NMR measurements and comparing normalised α-, β-, and γ-ATP resonances over the entire duration of the measurement. ATP levels remained stable over the measurement time for all experiments performed under standard cell culture conditions (Figure S2, [23]). After 6 h of measurement the ATP/Pi ratio decreased to 84% of the initial ratio.

### 2.2. ^1^H NMR of Cells and Medium without Separation in the Bioreactor for Protocol Optimisation and Metabolite Assignments

**Figure 2:**
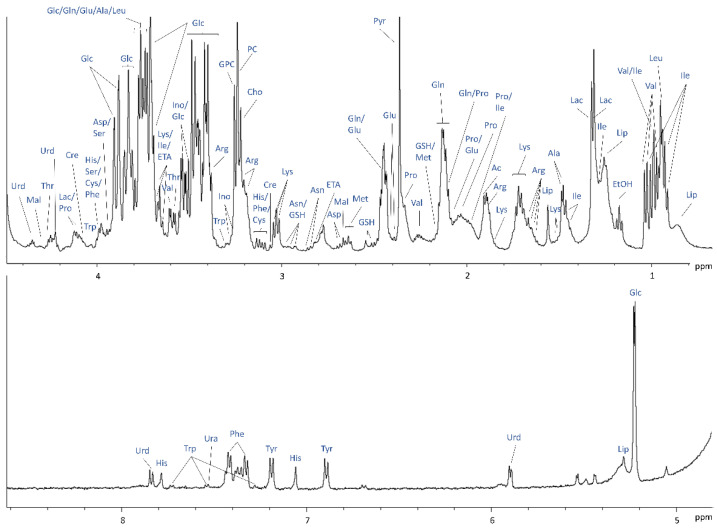
^1^H NMR spectrum of Cultrex embedded 10 × 10^6^ FBCO04 with assignments of selected metabolites identified.

^1^H NMR measurements of embedded cells in the bioreactor were performed with the rationale of improving spectral quality and increasing the signal-to-noise ratio (SNR) for additional peak assignments. Increased experience in scaffold preparation led to more homogeneous scaffolds, devoid of air bubbles resulting in improved shimming. Temporary elimination of PFTBA carriers from collagen-based scaffold preparation, which may cause a deterioration in NMR signal quality, also allowed the achievement of better magnetic field homogeneity, to the detriment of oxygen quantification. All these factors, plus the restriction of scaffold embedded cells to the sensitive region of the NMR coil by a gel plug placed at the bottom of the 5 mm NMR tube, led to an increase in spectral quality and SNR. ^1^H NMR spectra of 3D collagen-based matrix with 10 million fibroblasts were acquired under a flow rate of 0.1 mL min^−1^ and 35 metabolites were identified (Figure 2). A complete list of ^1^H NMR resonances, which were assigned to intra- and extracellular compounds, is summarised in Table S1. However, both intracellular and extracellular medium contributions were included in the ^1^H NMR spectra, complicating the interpretation of possible metabolic changes in the bioreactor.

### 2.3. Validation of Diffusion Technique and Quantification Method

**Figure 3:**
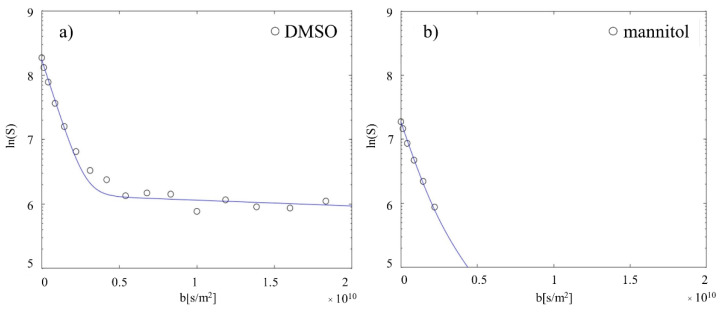
Substance spiking experiments. (**a**) 1 mM DMSO was spiked in the inlet and the diffusion behaviour characterised. As expected, DMSO shows a biexponential decay (extrac: 97.7 ± 1.0%; intrac: 2.3 ± 1.0%), being permeable to the cell membrane. (**b**) 0.25 mM of cell membrane impermeable mannitol was spiked in the inlet and the diffusion behaviour characterised. As expected, mannitol shows only a fast monoexponential decay, being localised only in the extracellular space. The same number of points were acquired for both measurements, but for mannitol the signal intensities reached the noise level (equal to 0) for higher b-values and cannot be displayed in the logarithmic plot.

Validation experiments described below were conducted in order to determine the reliability and sensitivity of the intra- and extracellular separation method using diffusion measurements. In two independent spiking experiments, 1 mM dimethyl sulfoxide (DMSO) and 0.25 mM mannitol were added to the perfused cell culture medium at the bioreactor’s inlet and their diffusion behaviour was characterised. The rationale was to see if the diffusion measurements of these two compounds were able to reflect their properties: DMSO has been shown to cross the cellular membranes [24,25]. On the other hand, mannitol is not able to cross phospholipid bilayers, being impermeant and non-metabolised by cells [26]. Accordingly, the measured signal attenuation of DMSO was clearly biexponential (Figure 3a), showing that DMSO was present in both compartments, with an expected low intracellular DMSO concentration for 3D collagen-based matrix embedded 10 million fibroblasts (2.3% of the total DMSO concentration in the NMR tube). Signal attenuation of mannitol, instead, was best described by a mono-exponential decay with one single fast slope and no slow component (Figure 3b). Therefore, the different diffusion behaviour for the two substances confirmed expectations, thus validating the application of diffusion techniques for intra- and extracellular contribution separation.

In order to examine the absolute quantification method of intra- and extracellular metabolites, in a first step pure cell culture medium was perfused in the system and diffusion behaviour of freely diffusing metabolites and water was studied. As expected, signal attenuations were described best by mono-exponential decays, and the absolute concentrations, calculated with the Electronic Reference To access In vivo Concentrations (ERETIC) method, showed a high correlation (R² = 0.989) with the known concentrations of metabolites contained in the cell culture medium (Table S2). Finally, the combination of the ERETIC quantification method with the diffusion separation method of intra- and extracellular contributions was examined in independent measurements on collagen embedded fibroblasts perfused with different cell culture media as described below.

### 2.4. ^1^H NMR Diffusion Measurements

In order to separate intra- and extracellular contributions in NMR spectra obtained within the Bioreactor, perfusion measurements (I) with cell culture medium with high amino acids (AA), (II) with pure phosphate-buffered saline (PBS) supplied with glucose, glutamine, and pyruvate, and (III) with standard culture medium (Table S3) were performed separately in independent experiments on 3D collagen-based matrix with 10 million fibroblasts and diffusion behaviours of 19 intra- and extracellular metabolites and water were studied.

**Figure 4:**
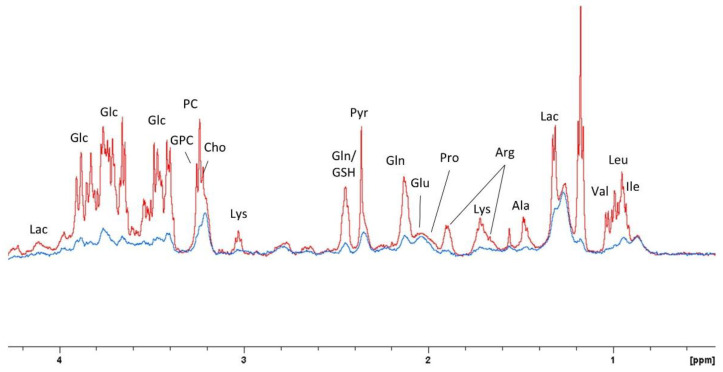
1D overlapped spectra extracted from pseudo 2D DOSY measurement with resonance assignments at 6.8 mT/m (b = 8.65 × 10^6^ s m^−2^, in red) and 149.9 mT/m (b = 4.19 × 10^9^ s m^−2^, in blue).

Representative 1D overlapped diffusion spectra at low and high b values are represented in Figure 4. At low b-factor values the SNR was high and both intra- and extracellular metabolites contributed to the NMR signal intensities. At b-values higher than approximately 4.0 × 10^9^ s m^−2^, primarily intracellular signal contributions were measured for all metabolites and water.

**Figure 5:**
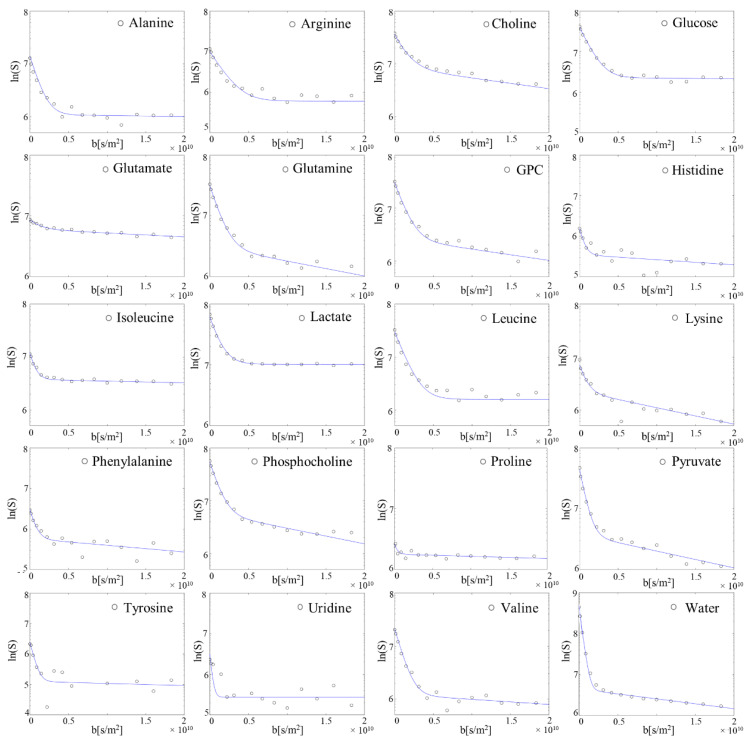
Decay curves from a representative single measurement of metabolite and water signal intensities as a function of diffusion-weighting factor b with biexponential fitting (solid lines). All observed 19 metabolites and water show a biexponential decay, being distributed in both intra- and extracellular compartments.

The logarithm of signal attenuations as a function of diffusion-weighting factor b is displayed in (Figure 5) for standard cell culture conditions. Attenuation curves for metabolites and water were well described by a biexponential fitting model, indicating the presence of two predominant compartments. Signal attenuation of metabolites located in the extracellular space was obtained at smaller b-values (b < 0.5 × 10^10^ s m^−2^), and its decay was described by a diffusion constant D_fast_ (approx. 10^−9^ m^2^ rad^−1^ s^−1^, Table 1). At higher b-values, instead, lower signal attenuation of compartmentalised molecules was described by two orders of magnitude reduced diffusion constant D_slow_ (approx. 10^−11^ m^2^ rad^−1^ s^−1^, Table 1). The intercepts of fast and slow slopes were used to calculate the intra- and extracellular distribution of metabolites and water (as described in [9]), expressed in percentage in Table 1. ERETIC signals were used to convert these percentage values into absolute concentrations (Table 1).

Table S3 shows the quantitative intra- and extracellular results of the additionally performed diffusion measurements on embedded cell cultures perfused with media containing different levels of amino acids. The results reflect the different perfusion conditions. During perfusion with pure PBS only supplemented with glucose, glutamine, and pyruvate, the estimated concentrations of most AA were lower both intra- and extracellularly than in both normal condition and cell culture medium with high AA, except for the supplemented metabolites. Determined concentrations of glucose, pyruvate, and glutamine in the extracellular space were comparable for normal, high AA medium and supplemented PBS perfusion conditions. During perfusion with medium at high amino acids concentration, both intra- and extracellular estimated concentrations of most AA were higher than in the other conditions.

The water diffusion behaviour was also investigated. The fast slope due to diffusion of extracellular water molecules was obtained at smaller b-values (b < 0.2 × 10^10^ s m^−2^), yielding a diffusion constant D_fast_ = 3.2 × 10^−9^ m^2^ rad^−1^ s^−1^, corresponding approximately to the known diffusion constant of free water at 310 K [16,27,28,29]. Attenuation signal of compartmentalised water molecules, instead, was described by a diffusion constant D_slow_ = 6.3 × 10^−11^ m^2^ rad^−1^ s^−1^. We found that only 1.0% of total water in the sample volume in the sensitive NMR region was compartmentalised within cell membranes. Assuming that in fibroblasts, water makes up 70% of the total cellular volume [30], we estimated that 1.4% of the total sample volume was occupied by cells, in excellent agreement with the results found with ^23^Na NMR. An additionally performed water diffusion measurement on 3D collagen-based matrix with 20 million fibroblasts revealed correspondingly that 3.2% of total sample volume was occupied by cells.

### 2.5. Glycolytic Stress Test

Previous measurements demonstrated the sensitivity of the cell culture bioreactor system to detect metabolic changes during constant perfusion upon inhibitor challenge, however without separating intra- and extracellular compartments. Based on these measurements, a simplified glycolytic stress test was performed, and diffusion behaviour of metabolites and water was studied.

**Figure 6:**
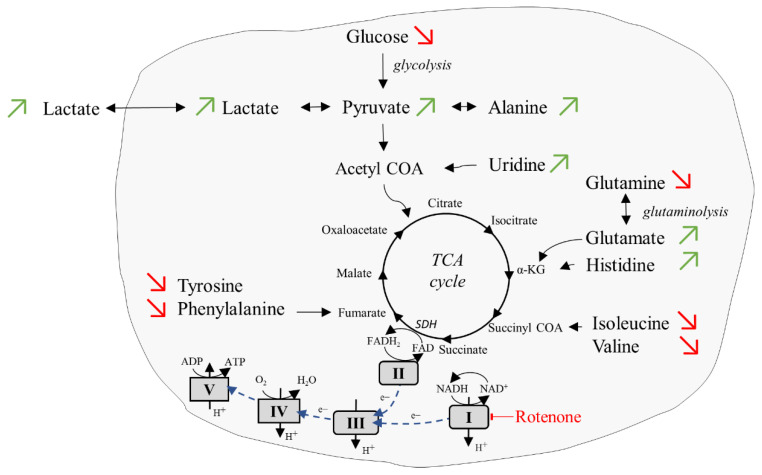
Cellular respiration upon addition of inhibitor rotenone. In the inhibition condition, an upregulation of the glycolytic activity and an adaptation to complex-II dependent respiration were observed. Red arrows: substrates reduced compared to basal condition. Green arrows: substrates increased compared to basal condition.

The addition of rotenone, an inhibitor of the mitochondrial respiration chain, led to strong changes of intra- and extracellular amino acid concentrations (Table 2, Figure 6). An increase in lactate (0.77 to 0.97 mM) in the extracellular space, together with the simultaneous decrease in glucose (36.7 to 20.7 mM) and increases of pyruvate (2.5 to 4.7 mM) and lactate (19.2 to 24.4 mM) in the intracellular space were detected confirming our previous study where these changes had been related to an upregulation of glycolytic activity [8]. The finding of an increase in intracellular alanine concentration (0.67 to 2.5 mM) may be associated with the conversion of high pyruvate and glutamate levels via transamination reaction. The simultaneous decrease in intracellular glutamine (18.4 to 8.5 mM) and increase in intracellular glutamate (15.6 to 16.6 mM) was associated with an ongoing glutaminolytic activity. Indeed, we noticed increases in all intracellular metabolites (glutamate, histidine, leucine, uridine) feeding the tricarboxylic acid cycle (TCA) cycle up to the rotenone blockade of complex I (CI) enzyme of the electron transport chain (Figure 6). On the other hand, we observed decreases in intracellular metabolites (i.e., isoleucine, valine) feeding the respiratory chain through Complex II (CII), independent of CI-dependent respiration, showing a possible residual ATP production via succinate dehydrogenase (SDH), which participates in the TCA cycle, but it is also an integral part of the electron transport chain.

### 2.6. Supernatant Collection and Extracellular Footprint Determination

**Figure 7:**
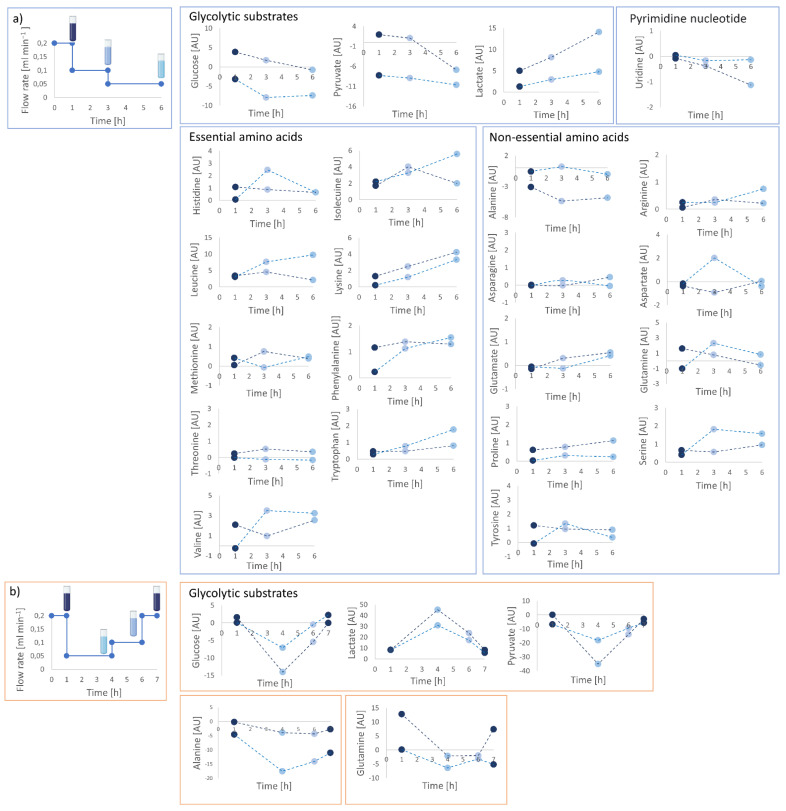
Calculated extracellular footprints via spectral subtraction of the pure cell medium spectrum from the out-flowing supernatant spectra collected at different flow rates under applied flow rate sequences of (**a**) 0.2–0.1–0.05 mL min ^−1^ and (**b**) 0.2–0.05–0.1–0.2 mL min^−1^. Reversible changes in metabolite levels were observed at the different applied flow rates. Two separate measurements were performed for both flow rates and are displayed individually connected via dashed lines. Different nominal values of highly concentrated metabolites (i.e., glucose, pyruvate, lactate) may be associated to different cell number due to cell count.

In order to investigate the sensitivity to determine the metabolic footprint of the cells and metabolic variations, supernatant samples were collected from the bioreactor’s outlet at different flow rates. In an initial attempt, a sequence of 0.2–0.1–0.05 mL min^−1^ was applied in two independent measurements of 3D collagen-based matrix with 10 million fibroblasts, and supernatant samples were collected at each applied flow rate. The rationale was that different flow rates will lead to different accumulations of produced and reductions in consumed metabolites. A control cell culture medium sample was collected before the measurement. ^1^H NMR spectra were acquired, and extracellular footprints were determined via spectral subtraction of the pure cell medium spectrum from the outflowing supernatant spectra. The results demonstrate the sensitivity of the cell culture bioreactor system to detect metabolic changes in the collected supernatant samples at different flow rates over time. Lactate production and glucose, pyruvate, alanine, and uridine consumption could be monitored at all applied flow rates (Figure 7a). For the highest flow rate of 0.2 mL min^−1^, the lowest ratio of lactate production to glucose consumption was detected, whereas higher ratios were measured at lower flow rates. The concurrent highest glutamine consumption and glutamate production were detected at flow rate of 0.05 mL min^−1^. As the flow rate decreased, increasing alanine and uridine consumption were measured. Increase in alanine consumption may be associated with alanine conversion into pyruvate and glutamate via transamination reaction, to sustain both TCA cycle and glutaminolytic pathways. Increasing uridine consumption, instead, could be explained with uridine catabolism into acetyl CoA, to feed the TCA cycle [31].

In order to see if the cells were not irreversibly damaged due to low flow, and if there was an effect due to the measurement duration, two additional independent experiments with a flow rate sequence of 0.2–0.05–0.1–0.2 mL min^−1^ were performed on a perfused 3D scaffold with 10 million fibroblasts, and supernatant samples were collected. Extracellular footprints determination was performed and demonstrated that produced and consumed metabolite levels at specific flow rates were not dependent on the sequence applied (Figure 7b). The analysis of glycolytic substrates and alanine revealed reversibility of metabolic changes, returning to initial metabolic levels at the same applied flow rate (i.e., 0.2 mL/min), thus confirming that detected changes were not due to reduced cell viability.

## 3. Discussion

In this study, we showed the possibility of the cell culture bioreactor system to perform in real-time separated intra- and extracellular quantitative metabolic analysis of human control fibroblasts. We optimised the scaffold preparation procedure, and we performed a complete 3D cell culture characterisation. We determined scaffold volume and shrinkage by using microscopy analysis and ^23^Na NMR, and we evaluated the cellular volume by employing water diffusion and ^23^Na measurements. We used diffusion weighted NMR spectroscopy to separate between intra- and extracellular metabolites and determined absolute metabolite concentrations. The reliability and sensitivity of the method to detect concentration changes in both intra- and extracellular metabolites was shown. Estimated intra- and extracellular amino acid pools in perfusion experiments with selective cell culture media reflected the medium compositions, varying depending on supplemented media metabolites. Amino acid concentration changes upon rotenone addition were detected in both intra- and extracellular compartments. We also showed the capability to determine the metabolic footprint of the cells by collecting supernatant samples from the bioreactor’s outlet, and to determine metabolic variations at different flow rates. Compared to the extracellular metabolite determination within the bioreactor using the diffusion separation procedure, the metabolic determination using the outflow provides complementary information because of improved spectral quality of the NMR spectra due to non-overlapping resonances from intracellular contributions and because the measurements could be performed without media flow.

The bioreactor system had been already established and tested in our previous study [8], where different cell numbers and flow rates were evaluated to set up the optimal conditions for real-time NMR investigation of living perfused cells. High cell numbers lead to a strong intracellular NMR signal and fast detectable metabolic changes, however, at the cost of reduced spectral quality. On the other hand, low cell number lead to a higher spectral quality, but weaker intracellular NMR signal and slower detectable metabolic changes. For this reason, we chose an intermediate cell number to compensate for both side effects.

3D collagen-based matrix embedded 10 million fibroblasts led to an expected sample volume reduction of 56% after encapsulation in the first 20 h and remained stable afterwards. ^23^Na NMR measurements were performed in order to investigate if this reduction in volume was due to either degradation of 3D collagen-based matrix in cell culture incubator condition or matrix remodelling by fibroblasts. The detection of similar sodium levels in the first 24 h (Figure S1), indicates that the strong decrease in the scaffold volume is associated with a fibroblasts contractile action that causes an increase in polymer chain tension and subsequent medium leakage from 3D matrix porosities (i.e., “squeezing effect”). This effect was described by [32,33], who discussed collagen-based matrix size reduction by development of reciprocal mechanical interactions with adherent fibroblasts.

^23^Na NMR measurements were also used to establish the cell volume, which was found to be small compared to the total sample volume within the NMR sensitive region of the bioreactor tube. This non-invasive method for cell volume determination in a perfused sample was already shown by Mancuso et al. [20], who emphasised its higher reliability and precision for cell volume assessment compared to other techniques based on nutrient uptake rates (i.e., oxygen, total intracellular NTP) estimations.

Despite the small cell volume in the NMR sample, we have already shown the possibility of the bioreactor system coupled with NMR spectrometer to study cellular metabolism [8], but without discerning between intra- and extracellular contributions. The separation presented in this study represents to our knowledge a new approach of performing intra- and extracellular metabolic analysis, since until now studies of intracellular metabolic fingerprinting have been mainly performed on lysed cells and cell suspensions providing metabolic snapshots [1,34]. Hertig et al. [1] performed both intracellular fingerprinting and extracellular footprinting analysis using NMR spectroscopy on lysed cells and extracellular cell culture medium, respectively [1]. The untargeted fingerprinting NMR analysis revealed unique physiological insight on metabolic adaptations in pyruvate dehydrogenase (PDH) and complex I deficient fibroblasts under selective culture conditions. ^1^H NMR intracellular fingerprint and extracellular footprint analyses were also performed in [35], to study the metabolic response of Escherichia coli cultures to different antibiotics. They highlighted how these complementary analyses provided information on intracellular and extracellular targets, such as protein, DNA and cell wall. Czajkowska et al. [36] employed ^1^H NMR extracellular footprinting analysis to investigate in vitro interactions between S. aureus biofilm and fibroblasts. However, all these studies were performed as single measurements.

In this study, we showed the sensitivity to detect metabolic changes in the collected supernatants from the bioreactor’s outlet, by simultaneous monitoring metabolites secreted into, and consumed from the perfused cell culture medium. The advantages of this method are the higher NMR spectral quality of the liquid samples compared to the spectra in the bioreactor, the absence of membrane lipids contribution which may overlap with other resonances, the minimal sample preparation, and the possibility to monitor longitudinally metabolic changes of living cells due to substrates or inhibitors challenges. Supernatant measurements, in fact, were widely performed by MS [37], and also by NMR [38], but rarely in NMR bioreactor systems for longitudinal real-time metabolic investigations [39,40].

Besides extracellular footprint determination from the outflow, which is complementing the separation of compartments within the bioreactor, we investigated both intra and extracellular fractions by analysing the diffusion properties of metabolites and water on 3D collagen-based matrix embedded fibroblasts. Diffusion-weighted NMR spectroscopy has been widely used in perfused cell culture systems to separate intra- and extracellular contributions of water and very rarely to quantify the intracellular contribution of a few metabolites [9,16,27,41,42,43]. In this study, diffusion measurements of water revealed a small intracellular water fraction and, by assuming that water represents 70% of the total volume in fibroblast [30], we found a low cell volume and a low ratio of cells to extracellular medium that matched very well the ratio obtained by sodium NMR. Investigation of the diffusion of metabolites led to intra- and extracellular separation of 19 metabolites. Most of the determined extracellular absolute concentrations agreed with concentration values of metabolites in the perfused cell culture medium (Table S2). In measurements with cells only glucose, pyruvate, and glutamine concentrations were estimated lower than expected in the bioreactor tube (Table S3), which however may be explained as they are the main substrates of two mitochondrial fuel pathways such as glycolysis and glutaminolysis. Intracellular concentration levels of alanine, glutamate, isoleucine, leucine, phenylalanine, tyrosine, and valine were instead comparable with values found for human foreskin fibroblasts cultivated in normotonic condition [44].

We showed the dependence of intra- and extracellular metabolite concentration levels on selective perfused culture media (Table S3). Changes in the intra- and extracellular metabolite pool concentrations were also obtained upon the addition of the respiratory chain inhibitor rotenone. In this case, we observed a simultaneous decrease in intracellular glucose and an increase in intracellular lactate and pyruvate, indicating an upregulation of glycolysis. We have already shown previously an upregulation of glycolytic activity and an increase in measured oxygen concentration in the bioreactor tube upon the addition of respiratory chain inhibitors oligomycin and rotenone [8], however, without separation of intra- and extracellular contributions. Unfortunately, in the current study oxygen was not quantified thus limiting interpretation of substrate changes directly linked with the TCA cycle and mitochondrial OXPHOS. However, an adaptation was observed of cellular metabolic respiration from CI-dependent oxidation of NADH toward CII-dependent respiration, since the simultaneous increase in intracellular glutamate, histidine, and uridine, which feed the respiratory chain through CI-dependent respiration, and the decrease in intracellular isoleucine, valine, phenylalanine, and tyrosine, which feed the respiratory chain through CII and SDH-dependent respiration, were detected (Figure 6).

The inclusion of oxygen measurements as performed before [8] are anticipated for future studies, to monitor the interdependence between cellular metabolism and energy production. Studies on other different metabolically active cell lines with the NMR bioreactor may be performed, using the appropriate extracellular matrices and cell culture conditions.

## 4. Materials and Methods

### 4.1. From 2D to 3D Cell Culture

#### 4.1.1. 2D Cell Culture

Human fibroblasts (FBCO04) have been obtained from skin biopsy from healthy controls in routine diagnostics and supplied to the routine cell culture laboratory of the Center of Laboratory Medicine of the University Hospital Bern. The cells were cultivated in 2D as carried out previously in [8]. In short, the control fibroblast cell line was cultured in MEM supplemented with 10% foetal calf serum, 1× non-essential amino acids (100 μM glycine, L-alanine, L-asparagine, L-aspartic acid, L-glutamic acid, L-proline, L-serine) (GIBCO/Invitrogen, Carlsbad, CA, USA), 2 mM L-glutamine, 200 μM uridine, 1 mM sodium pyruvate, 100 U mL^−^1 penicillin, and 100 μg mL−1 streptomycin and 10 μg mL^−1^ chlortetracycline at 37 °C and 5% CO_2_. The medium was exchanged between two and three times a week. Fibroblasts were cultured at 37 °C in a humidified 5% CO_2_ cell culture incubator and were passaged using 0.05% trypsin–EDTA. Cell numbers were determined in a Neubauer hemacytometer using the trypan blue exclusion method.

#### 4.1.2. 3D Cell Culture Preparation

Cell suspensions were obtained by harvesting roughly 80% confluent adherent human fibroblasts using 0.05% trypsin-EDTA. Cells were centrifuged (2000 rcf, 5 min) and resuspended in cell culture medium. Cell number was determined in a Neubauer hemacytometer and cell pellet containing the desired cell number was centrifuged (2000 rcf, 5 min) for the following embedding procedure. Cell pellet was diluted in 500 µL cell culture medium and 500 µL liquid collagen-based membrane matrix (Cultrex^TM^ RGF BME Type R1) and subsequently polymerised at 37 °C inside a sterile infusion tube (Volumed^®^-Set Silicone). After polymerisation, the 3D scaffold was gently released into a Petri dish containing 35 mL medium and incubated for 24 h [8].

### 4.2. Preparation for the NMR Measurement

In order to prevent embedded cells to settle at the bottom of the NMR tube outside the sensitive volume of the probe, a ~1 cm-high bottom plug was created by mixing 80 µL liquid collagen based Cultrex with 20 µL cell culture medium and letting it polymerise at the bottom of the tube. Subsequently, the 3D scaffold was taken up from the Petri dish using an infusion tube and inserted into the InsightMR^TM^ glass tube. The glass tube containing the 3D scaffold, placed on the upper surface of the gel plug, was gently connected to the tube of the bioreactor (see below) and placed within the NMR spectrometer.

### 4.3. NMR Bioreactor Setup

NMR experiments were performed on a 500.13 MHz Bruker Avance II spectrometer (Bruker BioSpin, Billerica, MA, USA). The instrument is equipped with a 5 mm ATM BBFO probe with z-gradient that was used for high resolution NMR experiments of cell culture samples. A cell culture bioreactor system within the NMR spectrometer, was employed and established previously in [8]. It makes use of a flow tube (InsightMR^TM^, Bruker) and its further specialisation InsightCell^TM^. The bioreactor was perfused using MEM supplemented with 1× non-essential amino acids (100 µM glycine, L-alanine, L-asparagine, L-aspartic acid, L-glutamic acid, L-proline, L-serine) (GIBCO/Invitrogen), 2 mM L-glutamine, 200 µM uridine, 1 mM sodium pyruvate, 100 U mL^−1^ penicillin, 100 µg mL^−1^ streptomycin and 10 µg mL^−1^ chlortetracycline, and 5% D2O. In selected samples different medium compositions were used, and glycolytic stress tests (*n* = 2) were performed by the addition of rotenone 5 µM (CI inhibitor) in the perfusion system, in order to inhibit the mitochondria oxidative phosphorylation. All experiments were carried out at 37 °C and 5% CO_2_.

### 4.4. Determination of NMR Sensitive Region

NMR sensitive region was determined in order to optimise the signal-to-noise ratio by placing the entire 3D cell culture sample within it and for calculation of the volume ratio between medium and cells (see below). The sensitive volume was found by determining the tube profile (using “calibgp”, Bruker pulse-program library).

### 4.5. Determination of Scaffold Volume

3D Collagen-based scaffolds (*n* = 4) were realised in parallel, by embedding 5, 10, and 20 million fibroblasts, and cultured in standard cell culture incubator up to 24 h (Figure S1). For each cell number, a sample per time point (1, 6, 20, and 24 h) was observed with optical microscope in order to estimate the scaffold volume, and then measured with NMR for ^23^Na evaluation. The scaffold volume was calculated by considering the sample as a cylinder, estimating both axial and radial directions with the digital image processing software GIMP 2.10.22.

^23^Na NMR measurements were performed for quantification of the ratio extracellular medium to collagen-based matrix in the sensitive NMR region of the bioreactor tube. Considering that sodium contribution was determined by medium and cells, and excluding intracellular sodium losses through cell membrane ruptures from LDH quantification, we postulated that changes in the detected sodium level were due to variations of the ratio medium to scaffold volume. ^23^Na NMR spectra were measured at nominal temperature of 37 °C, a spectral width of 13,000 Hz, a data size of 8 K points, 32 scans, and an acquisition time of 0.31 s. The total experiment time was 11 s. The free induction decays (FIDs) were exponentially weighted with a line broadening factor of 20 Hz, Fourier-transformed, manually phased and baseline corrected.

### 4.6. ^1^H NMR for Metabolic Analysis

Each ^1^H NMR spectrum was acquired using the 1D PROJECT pulse sequence with water presaturation and a T2-filter of 80 ms [45]. Each 1D ^1^H NMR spectrum was measured using the same parameters: a nominal temperature of 37 °C, a spectral width of 6000 Hz, a data size of 32 K points, 64 number of transients, an acquisition time of 2.73 s, and a relaxation delay of 4 s. The total experiment time for the ^1^H NMR acquisition was about 7 min. The spectral processing was performed using the Bruker Topspin software (version 3.2, patch level 5). The FIDs were exponentially weighted with a line broadening factor of 1 Hz, Fourier-transformed, manually phased and baseline corrected and calibrated according to the left peak of the lactate -CH3 doublet (1.324 ppm). Spectral peak assignments were based on our previous assignments [1,8] and supported by literature references [46,47], an in-house reference data bank, and the Human Metabolome Database (HMDB) [48].

^1^H spectral buckets were manually selected and integrated based of their variable peak width.

### 4.7. ^1^H NMR for Diffusion Measurements

^1^H NMR diffusion NMR measurements (*n* = 6) were performed using a ‘pseudo’ 2D stimulated echo experiment with bipolar gradient pulses and a longitudinal eddy current delay (“ledbpgp2s2d”, Bruker pulse-program library), further modified by substitution of the ‘cpmg’ with the ‘PROJECT’ block. The measurements were performed both with and without presaturation of the water signal, to measure the diffusion decay of metabolites and water signal, respectively. Each 2D ^1^H diffusion acquisition was measured applying an intermediate flow rate of 0.1 mL min^−1^, in order to avoid flow artefacts in the extracellular diffusion components [16], at nominal temperature of 37 °C. The selected diffusion time Δ was 83 ms, and the gradient pulse length δ was 6 ms. The diffusion-weighting factor b was increased from 8.65 × 10^6^ up to 1.83 × 10^10^ s m^−2^ by applying linear ramps of 16 different values of sinusoidal shaped gradient pulses, from 0.68 to 31.33 G cm^−1^. For each acquired spectrum of the 2D-diffusion pulse sequence, a data size of 32 K points, a number of 32 transients, and an acquisition time of 2 s, were used.

The 2D-diffusion measurements were processed using Bruker Dynamics Center 2.6.3. Peaks intensities were determined from the spectra of the pseudo 2D acquisition and fitted using a biexponential model. A home-written MatLab program (R2020b, The MathWorks Inc., Natick, MA, USA) was used for fitting diffusion data and calculating diffusion parameters. The Electronic Reference To access In vivo Concentrations [49] method was used for absolute quantification of intra- and extracellular metabolites concentrations, by using 1 mM DMSO as external standard.

### 4.8. Supernatant Collection and Extracellular Footprint Determination

Out-flowing media (i.e., supernatants) from the outlet were continuously collected at exactly defined time intervals and at different flow rates (0.2, 0.1, and 0.05 mL min^−1^) in falcon tubes and stored at −80 °C in order to quench metabolic and enzymatic activity. Collected supernatant samples were subsequently measured in normal NMR glass tubes. Each supernatant ^1^H NMR spectrum was acquired using the same parameters of ^1^H NMR measurements on cells (Section 4 and Section 4.6), but at a nominal temperature of 2 °C. Extracellular footprints were determined via spectral subtraction of the pure cell medium ^1^H NMR spectrum (that was measured separately) from the out-flowing ^1^H NMR supernatant spectra (*n* = 2, per applied flow rate sequence).

### 4.9. ^31^P and ^23^Na NMR Measurements

^31^P NMR measurements were performed for energy status determination. All measurements were performed using a 1D sequence with F2 decoupling employing p0 for any flip angle (“zg0dc”, Bruker pulse-program library) at a nominal temperature of 37 °C, a spectral width of 10,000 Hz, a data size of 16 K points, number of transients 1024, an acquisition time of 0.81 s, and a relaxation delay of 100 ns. The total experiment time for the ^31^P NMR acquisition was about 15 min. The FIDs were exponentially weighted with a line broadening factor of 20 Hz, Fourier-transformed, manually phased and baseline corrected and calibrated according to the α-ATP signal to −11.30 ppm [50]. All ^31^P chemical shifts were assigned according to literatures references [51,52,53,54,55].

^23^Na NMR measurements were performed for calculation of the cell volume compared to the medium volume [20], by considering sodium concentration differences between intra- and extracellular compartments. All measurements were performed using a 1D sequence (“zg”, Bruker pulse-program library) at a nominal temperature of 37 °C, a spectral width of 13,000 Hz, a data size of 8 K points, number of transients 32, and an acquisition time of 0.31 s. For each sample, we assumed an intracellular sodium concentration of 10 mM, excluding possible losses though cell membrane ruptures from LDH quantification, and this absolute concentration was then converted into absolute integral value by using the integral of the extracellular medium, measured separately, with a known concentration of 158 mM sodium. Assuming a negligible intracellular contribution to the sodium level compared to the whole NMR sample, the gel fraction was calculated as follows:

F1(%) = ((Na_control medium_ − Na_sample NMR_)/Na_control medium_) × 100 (1)

The cell volume was estimated for each NMR sample by applying the equation reported in [20]. ^23^Na NMR spectra were measured as performed in Section 4.5.

## 5. Conclusions

In this work, we showed the possibility to perform with the NMR bioreactor system in real-time intra- and extracellular quantitative metabolic investigations of living cultured fibroblasts, combining a commercially available flow tube system with a standard 5 mm NMR probe. We performed a comprehensive 3D cell culture sample characterisation by evaluating scaffold volume and shrinkage, and by determining the cell volume and the ratio of cells to the extracellular medium within the sensitive region of the NMR coil. We revealed the capability of diffusion technique to detect changes in both intra- and extracellular metabolite pools under standard or selective culture condition, and upon inhibitor challenges. We showed the sensitivity to detect metabolite changes in the extracellular footprint at different flow rates. The methods described in this paper to longitudinally monitor cell-specific metabolic responses either in normal conditions or upon substrate challenges are of potential value for investigating defect fibroblasts for metabolomic characterisation of mitochondrial diseases and for improving physiological comprehension. The methods may provide a model for the evaluation of potential treatments, toxic effects, drugs, or inhibitor challenges also in other cell types.

## Figures and Tables

**Table 1:** Estimated diffusion constants from both fitted components and intra- extracellular distribution of 19 metabolites using a biexponential fitting model and estimated intra- and extracellular absolute concentrations using ERETIC signals. Representation of the mean ± SD of the values obtained from 6 independent experiments.

	D_fast_ ^1^	D_slow_ ^2^	Extra [%]	Intra [%]	Extra [mM]	Intra [mM]	Perfused MEM [mM]
Ala	1.78 ± 0.7	8.65 ± 4.4	93.72 ± 2.72	6.28 ± 2.72	0.24 ± 0.04	1.11 ± 0.39	0.1
Arg	1.72 ± 0.7	8.97 ± 7.6	90.13 ± 5.48	9.87 ± 5.48	0.68 ± 0.14	5.87 ± 4.44	0.6
Cho	1.10 ± 0.4	5.83 ± 1.6	78.50 ± 3.48	21.50 ± 3.48	0.11 ± 0.02	1.89 ± 0.14	-
Glc	1.24 ± 0.4	4.60 ± 4.4	92.57 ± 2.26	7.43 ± 2.26	5.1 ± 0.69	24.30 ± 7.74	5.55
Glu	0.80 ± 0.7	1.37 ± 1.0	46.50 ± 9.88	53.50 ± 9.88	0.16 ± 0.05	12.55 ± 3.34	0.1
Gln	1.43 ± 0.4	15.03 ± 8.0	86.97 ± 4.22	13.04 ± 4.22	1.23 ± 0.17	13.96 ± 6.38	1.95
GPC	1.39 ± 0.6	8.11 ± 6.5	90.54 ± 4.03	9.46 ± 4.03	0.11 ± 0.02	0.75 ± 0.38	-
His	1.85 ± 0.8	4.72 ± 5.7	86.18 ± 9.53	13.82 ± 9.53	0.11 ± 0.03	1.24 ± 1.01	0.2
Ile	1.67 ± 0.3	4.44 ± 3.9	87.38 ± 9.20	12.61 ± 9.20	0.31 ± 0.08	3.00 ± 2.32	0.4
Lac	1.24 ± 0.2	1.18 ± 0.6	80.44 ± 5.98	19.56 ± 5.98	1.17 ± 0.77	16.55 ± 4.43	-
Leu	1.23 ± 0.4	5.19 ± 5.1	94.17 ± 1.35	5.83 ± 1.35	0.33 ± 0.05	1.58 ± 0.7	0.4
Lys	1.47 ± 0.3	5.32 ± 3.3	86.90 ± 7.33	13.10 ± 7.33	0.32 ± 0.07	3.93 ± 2.89	0.4
PC	1.46 ± 0.5	8.95 ± 8.3	90.60 ± 2.76	9.40 ± 2.76	0.21 ± 0.04	1.60 ± 0.63	-
Phe	1.97 ± 0.6	5.90 ± 2.3	89.80 ± 6.45	10.20 ± 6.45	0.18 ± 0.04	1.33 ± 0.6	0.19
Pro	1.43 ± 0.9	2.22 ± 1.8	56.88 ± 10.47	43.12 ± 10.47	0.26 ± 0.08	13.66 ± 2.97	0.1
Pyr	1.67 ± 0.4	13.04 ± 5.1	92.11 ± 3.86	7.89 ± 3.86	0.51 ± 0.02	3.03 ± 1.22	0.98
Tyr	1.96 ± 1.0	4.07 ± 4.4	92.05 ± 4.20	7.95 ± 4.20	0.14 ± 0.02	0.90 ± 0.55	0.2
Urd	1.89 ± 0.6	3.10 ± 3.5	95.10 ± 3.46	4.90 ± 3.46	0.07 ± 0.02	0.23 ± 0.14	0.19
Val	1.60 ± 0.5	9.28 ± 9.3	91.25 ± 5.17	8.75 ± 5.17	0.40 ± 0.14	2.52 ± 1.36	0.39

^1^ (m^2^ rad^−1^ s^−1^) × 10^−9^; ^2^ (m^2^ rad^−1^ s^−1^) × 10^−11^.

**Table 2:** Estimated intra- and extracellular distributions and absolute concentrations of 14 metabolites in both basal and inhibition upon rotenone addition conditions.

Metabolite	Before Rotenone Addition (Basal Condition)				After Rotenone Addition (Inhibition Condition)			
	Extra [%]	Intra [%]	Extra [mM]	Intra [mM]	Extra [%]	Intra [%]	Extra [mM]	Intra [mM]
Ala	97.3	2.7	0.24	0.67	89.8	10.2	0.22	2.5
Glc	91.3	8.7	4.7	36.7	94.8	5.2	4.4	20.7
Glu	55.7	44.3	0.21	15.6	42.4	57.6	0.13	16.6
Gln	84.3	15.7	1.1	18.4	93.5	6.5	1.4	8.5
His	91.4	8.6	0.11	0.91	89.6	10.4	0.09	1.1
Ile	82.4	17.6	0.28	4.89	84.9	15.1	0.26	4.1
Lac	78.5	21.5	0.77	19.2	78.1	21.9	0.97	24.4
Leu	92.6	7.4	0.32	2.4	89.6	10.4	0.28	3.1
Lys	80.6	19.4	0.32	6.8	92.5	7.5	0.23	1.7
Phe	89.3	10.7	0.18	1.8	93.5	6.5	0.15	0.88
Pyr	95.2	4.8	0.53	2.5	90.5	9.5	0.5	4.7
Tyr	93.4	6.6	0.13	0.96	94.3	5.7	0.13	0.78
Urd	97.2	2.8	0.07	0.21	91.4	8.6	0.05	0.62
Val	89.7	10.3	0.31	3.4	90.8	9.2	0.26	2.6

## Data Availability

The datasets generated during and/or analysed during the current study are available from the corresponding author on request.

## Supplementary Materials

The following supporting information can be downloaded at: https://www.mdpi.com/article/10.3390/ijms23126555/s1.

## Abbreviations

MEM	Minimal Essential Medium
LDH	Lactate dehydrogenase
SNR	Signal-to-Noise Ratio
DMSO	Dimethyl sulfoxide
ERETIC	Electronic Reference To access In vivo Concentrations
AA	Amino Acids
PBS	Phosphate Buffered Saline
CI	Complex I
CII	Complex II
SDH	Succinate dehydrogenase
PDH	Pyruvate dehydrogenase
TCA	Tricarboxylic Acid Cycle
FBCO04	Control Fibroblasts N. 04
FID	Free Induction Decay
EDTA	Ethylenediaminetetraacetic acid
