# Supplementary material for: Determination of Intra- and Extracellular Metabolic Adaptations of 3D Cell Cultures upon Challenges in Real-Time by NMR
Source: Int J Mol Sci. 2022 Jun 12;23(12):6555. doi: 10.3390/ijms23126555 (PMC9223855; doi:10.3390/ijms23126555)
Supplement: Supplementary file 1 [file ijms-23-06555-s001.zip › ijms-1693722-supplementary.pdf]

Supplementary Materials

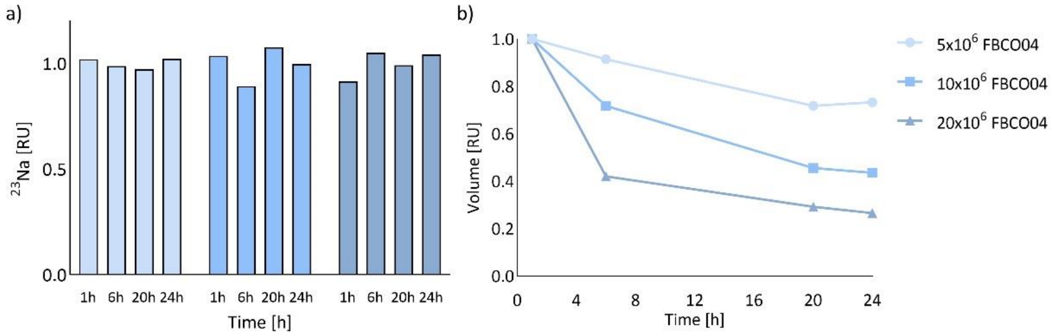

Figure S1. (a) Intensity of the ^{23}Na signal, in the first 24 h after sample realization, of 3D collagen-based matrix embedded 5, 10, and 20 million fibroblasts. Non-significant changes in sodium level revealed fibroblast mediated contraction rather than degradation of 3D collagen-based matrix in cell culture incubator condition before NMR measurements. Relative units (RU) were obtained from the normalization of each measurement to the average of all ^{23}Na measurements at all time points and for each cell density. (b) Scaffold volume reduction, in the first 24 h after sample realization, of 3D collagen-based matrix embedded 5, 10, and 20 million fibroblasts. As cell density increased, a larger volume contraction was observed. Relative units (RU) were obtained from the normalization to the first value (time point 1 h), for each cell density.

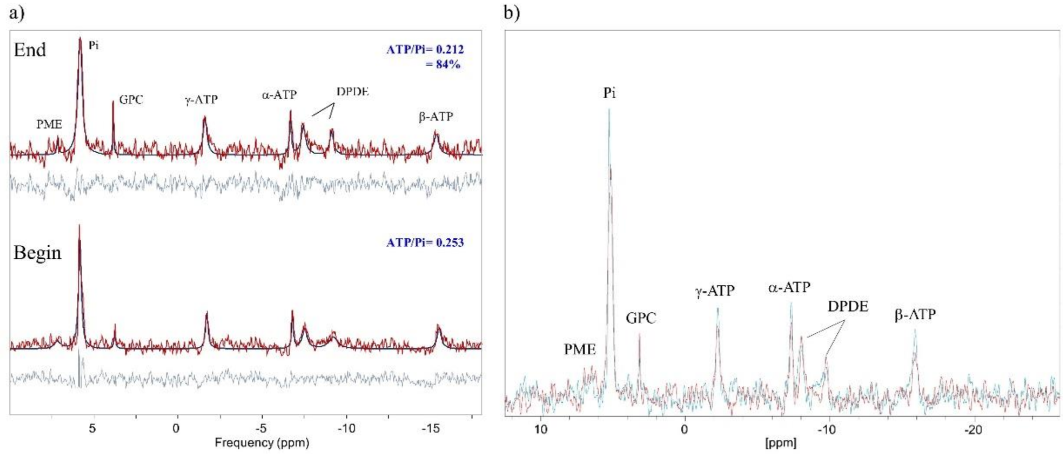

Figure S2. (a) ^{31}P spectra acquired at the beginning and at the end of a full-day experiment fitted with jMRUI [23]. (b) Overlapped ^{31}P NMR spectra with resonances assignments at the beginning (blue) and at the end (red) of a full-day experiment. ATP levels slightly decrease over the measurement time under standard cell culture conditions.

Table S1. Compounds identified in 1D PROJECTED ^{1}H NMR spectrum of perfused 3D collagen-based matrix embedded 10 × 10^6 FBCO04, with related chemical shifts (multiplicities) and number of protons per resonance.

Peak	Compound Name	^{1}H NMR Chemical shift [ppm], Multiplicity	Number of protons
1	Acetate (Ac)	1.928 (s)	3
2	Alanine (Ala)	1.478 (d), 3.791 (q)	3, 1
3	Arginine (Arg)	1.649 (m), 1.894 (m), 3.219 (t), 3.389 (t)	2, 2, 2, 1
4	Asparagine (Asn)	2.845 (m), 2.929 (m), 3.84 (m)	1, 1, 1
5	Aspartate (Asp)	2.667 (m), 3.93 (m)	2, 1

6	Choline (Cho)	3.218 (s), 3.53 (m), 4.07 (t)	9, 2, 2
7	Creatine (Cre)	3.04 (s), 3.935 (s)	3, 2
8	Cysteine (Cys)	3.073 (m), 3.70 (s), 3.979 (m)	2, 1, 1
9	Ethanol (EtOH)	1.174 (t), 3.651 (q)	3, 2
10	Ethanolamine (ETA)	3.13 (t), 3.83 (t)	2, 2
11	Glycerophosphocholine (GPC)	3.254 (s)	9
12	Glucose (Glc)	3.407 (m), 3.456 (m), 3.527 (dd), 3.738 (m), 3.827 (m), 3.888 (dd), 5.223 (d)	1, 1, 1, 3, 3, 2, 1
13	Glutamate (Glu)	2.095 (m), 2.14 (m), 2.416 (m), 3.769 (m)	2, 1, 1, 1
15	Glutamine (Gln)	2.121 (m), 2.442 (m), 3.759 (t)	2, 2, 1
16	Glutathione (GSH)	2.158 (m), 2.541 (q), 2.956 (t), 3.75 (m), 4.585	2, 2, 2, 3, 1
17	Histidine (His)	3.16 (q), 3.23 (q), 3.99 (t), 7.06 (s), 7.786 (s)	1, 1, 1, 1, 1
18	Isoleucine (Ile)	0.928 (t), 0.997 (d), 1.245 (m), 1.454 (m), 1.968 (m), 3.662 (d)	3, 3, 1, 1, 1, 1
19	Lactate (Lac)	1.324 (d), 4.104 (q)	3,1
20	Leucine (Leu)	0.94 (d), 0.95 (d), 1.714 (m), 3.739 (m)	3, 3, 3, 1
21	Lipid (Lip)	0.855, 1.256, 1.56, 5.285	-
22	Lysine (Lys)	1.46 (m), 1.715 (m), 1.846 (m), 3.027 (t), 3.658 (t)	2, 2, 2, 2, 1
23	Malate (Mal)	2.36 (dd), 2.66 (dd), 4.295 (dd)	1, 1, 1
24	Methionine (Met)	2.135 (m), 2.638 (t), 3.85 (t)	5, 2, 1
25	Phosphocholine (PC)	3.238 (s), 3.58 (m), 4.16 (m)	9, 2, 2
26	Phenylalanine (Phe)	2.84 (m), 3.117 (m), 3.983 (m), 7.327 (d), 7.372 (m), 7.416 (m)	1, 1, 1, 2, 1, 2
27	Proline (Pro)	2.001 (m), 2.080 (m), 2.345 (m), 3.345 (m), 3.427 (m), 4.137 (m)	2, 2, 1, 1, 1
28	Pyruvate (Pyr)	2.361 (s)	3
29	Serine (Ser)	3.84 (m), 3.971 (m)	1, 2
30	Threonine (Thr)	1.316 (d), 3.574 (d), 4.247 (m)	3, 1, 1
31	Tryptophan (Trp)	3.299 (q), 3.48 (q), 4.037 (m), 7.19 (m), 7.271 (m), 7.30 (s), 7.54 (d), 7.729 (d)	1, 1, 1, 1, 1, 1, 1, 1
32	Tyrosine (Tyr)	3.04 (q), 3.19 (q), 3.93 (q), 6.893 (d), 7.187 (d)	1, 1, 1, 2, 2
33	Uracil (Ura)	6.18 (d), 7.547 (d)	1, 1
34	Uridine (Urd)	4.236 (t), 4.348 (t), 5.904 (d), 7.847 (d)	1, 1, 3, 2
35	Valine (Val)	0.973 (d), 1.023 (d), 2.254 (m), 3.602 (d)	3, 3, 1, 1

Table S2. Comparison between absolute concentrations estimated with diffusion/ERETIC method and composition of 15 metabolites in perfused cell culture medium.

Metabolite	Diffusion/ERETIC [mM]	Medium [mM]
Ala	0.29	0.1
Arg	0.52	0.6
Glc	5.59	5.55
Glu	0.15	0.1

Gln	1.75	1.95
His	0.14	0.2
Ile	0.36	0.4
Leu	0.35	0.4
Lys	0.37	0.4
Phe	0.23	0.19
Pro	0.22	0.1
Pyr	0.53	0.98
Tyr	0.17	0.2
Urd	0.08	0.19
Val	0.37	0.39

Table S3. Estimated intra- and extracellular absolute concentrations of 19 metabolites in three different perfusion cell culture medium conditions (High AA medium (n=1, for method validation only); metabolic PBS supplied with glucose (Glc), glutamine (Gln), and pyruvate (Pyr) (n=1, for method validation only); normal cell culture medium (n=6)).

	**High AA medium**			**Glc, Gln, Pyr (buffered solution)**			**Normal cell culture medium**		
**Metabolite**	**NMR conc. Estimation**		**Medium [mM]**	**NMR conc. Estimation**		**Medium [mM]**	**NMR conc. Estimation**		**Medium [mM]**
	**Extra [mM]**	**Intra [mM]**		**Extra [mM]**	**Intra [mM]**		**Extra [mM]**	**Intra [mM]**	
Ala	0.33	0.82	-	0.03	0.29	-	0.24 ± 0.04	1.1 ± 0.4	0.1
Arg	0.85	13.0	0.5	0	0.10	-	0.68 ± 0.14	5.9 ± 4.4	0.6
Cho	0.08	5.5	-	0.08	2.3	-	0.11 ± 0.02	1.9 ± 0.1	-
GPC	0.10	1.5	-	0.08	1.5	-	0.11 ± 0.02	0.75 ± 0.38	-
Glc	4.7	44.1	5.55	5.1	15.5	5.55	5.1 ± 0.7	24.3 ± 7.7	5.55
Glu	0.11	11.9	-	0.11	7.1	-	0.16 ± 0.05	12.55 ± 3.3	0.1
Gln	1.6	9.6	1.95	1.5	13.0	1.95	1.2 ± 0.2	14.0 ± 6.4	1.95
His	0.18	1.7	0.3	0	0	-	0.11 ± 0.03	1.2 ± 1.0	0.2
Ile	0.65	5.2	0.8	0	0.29	-	0.31 ± 0.08	3.0 ± 2.3	0.4
Lac	0.99	26.2	-	0.23	13.4	-	1.2 ± 0.8	16.5 ± 4.4	-
Leu	0.64	4.7	0.8	0	1.1	-	0.33 ± 0.05	1.6 ± 0.7	0.4
Lys	0.69	3.5	1.0	0.11	1.5	-	0.32 ± 0.07	3.9 ± 2.9	0.4
PC	0.18	0.95	-	0.16	0.81	-	0.21 ± 0.04	1.6 ± 0.6	-
Phe	0.34	4.5	0.4	0	0.03	-	0.18 ± 0.04	1.3 ± 0.6	0.19
Pro	0.55	20.6	-	0.06	5.8	-	0.26 ± 0.08	13.7 ± 3.0	0.1
Pyr	0.59	4.4	0.98	0.45	3.4	0.98	0.51 ± 0.02	3.0 ± 1.2	0.98
Tyr	0.32	1.0	0.6	0	0.09	-	0.14 ± 0.02	0.9 ± 0.55	0.2
Urd	0.07	0.89	-	0	0	-	0.07 ± 0.02	0.23 ± 0.14	0.19
Val	0.68	1.5	0.8	0	0.02	-	0.4 ± 0.14	2.5 ± 1.4	0.39
